# Supplementary figures and images for: Apparent size and morphology of bacterial microcompartments varies with technique
Source: PLoS One. 2020 Mar 9;15(3):e0226395. doi: 10.1371/journal.pone.0226395 (PMC7062276; doi:10.1371/journal.pone.0226395)

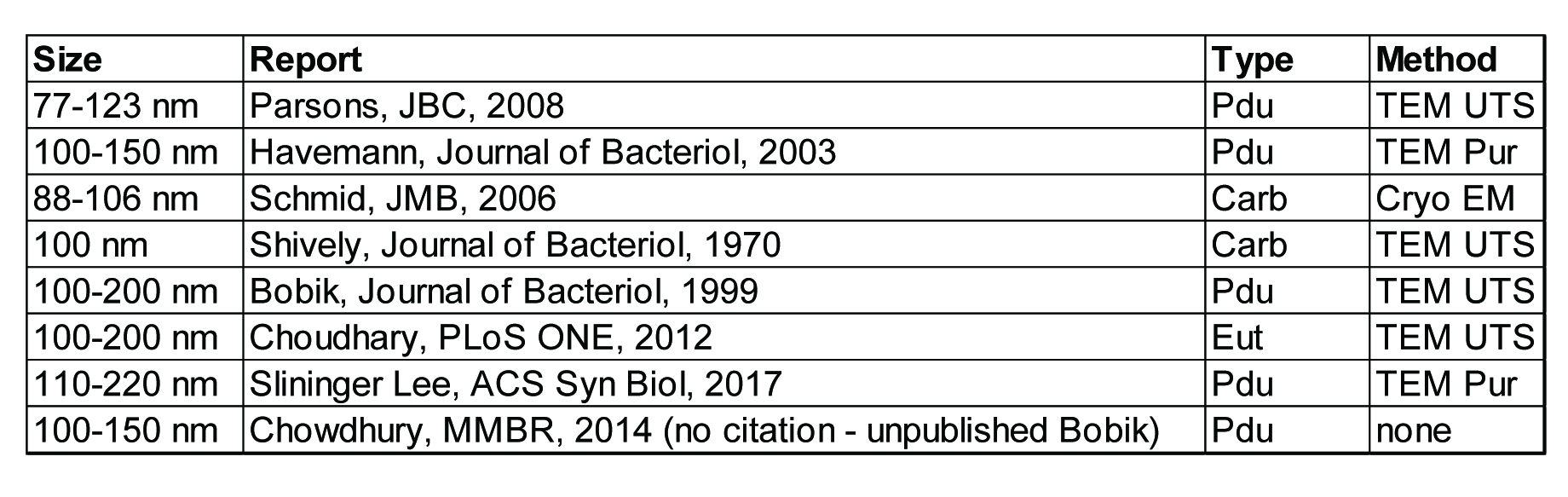

Supplement: S1 Table — The reported size range for MCPs, the work in which the size was reported, the system being analyzed (Propanediol utilization (Pdu), Ethanolamine utilization (Eut), Carboxysome (Carb)), and the technique used for the analysis (TEM of ultra-thin sections (TEM UTS), TEM of purified MCPs (TEM Pur)). (TIF) [file pone.0226395.s001.tif]

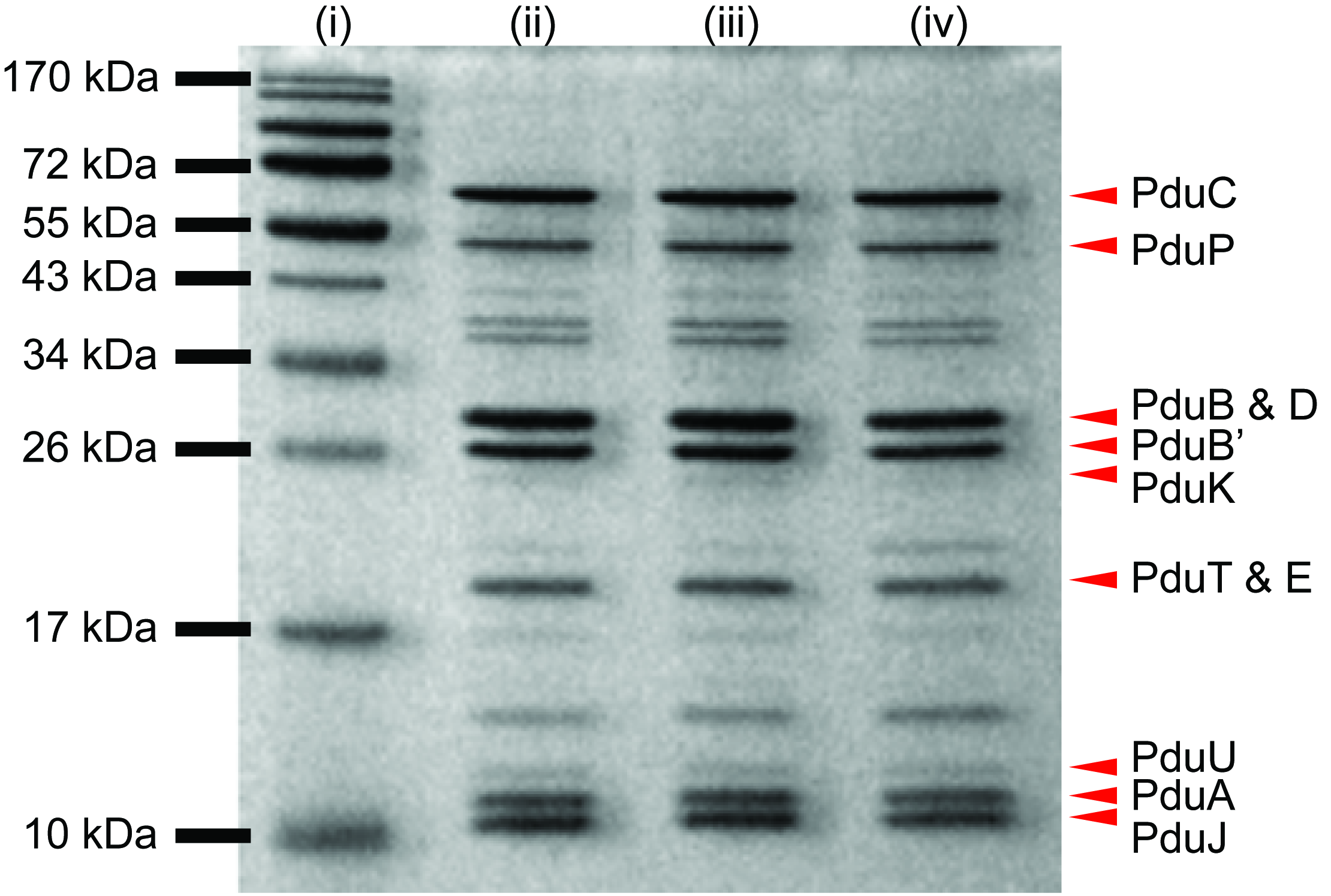

Supplement: S1 Fig — Lanes: (i) molecular weight standard, (ii-iv) replicates of purified Pdu MCPs. (TIF) [file pone.0226395.s002.tif]

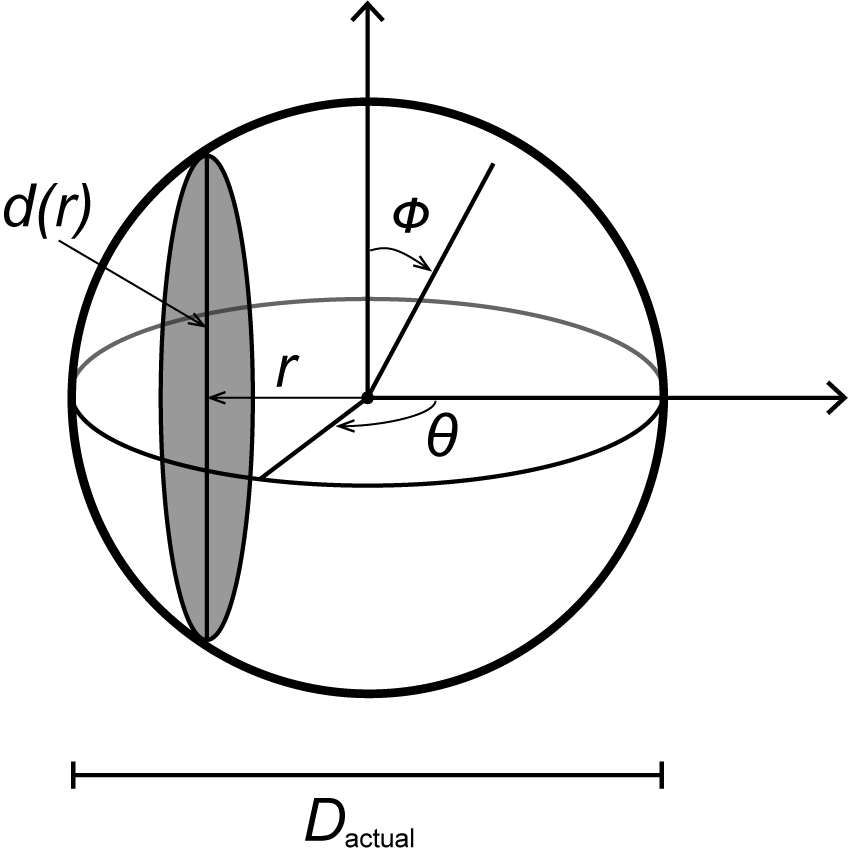

Supplement: S2 Fig — Diagram showing the parameters used in the calculation of the average diameter by ultra-thin sectioning. Dactual is the true diameter of the sphere, r is the variable describing the distance from the center of the sphere, θ is the azimuthal angle, Φ is the zenith angle, and d(r) is the diameter of an arbitrary circular slice in the sphere at distance r from the center. (TIF) [file pone.0226395.s003.tif]

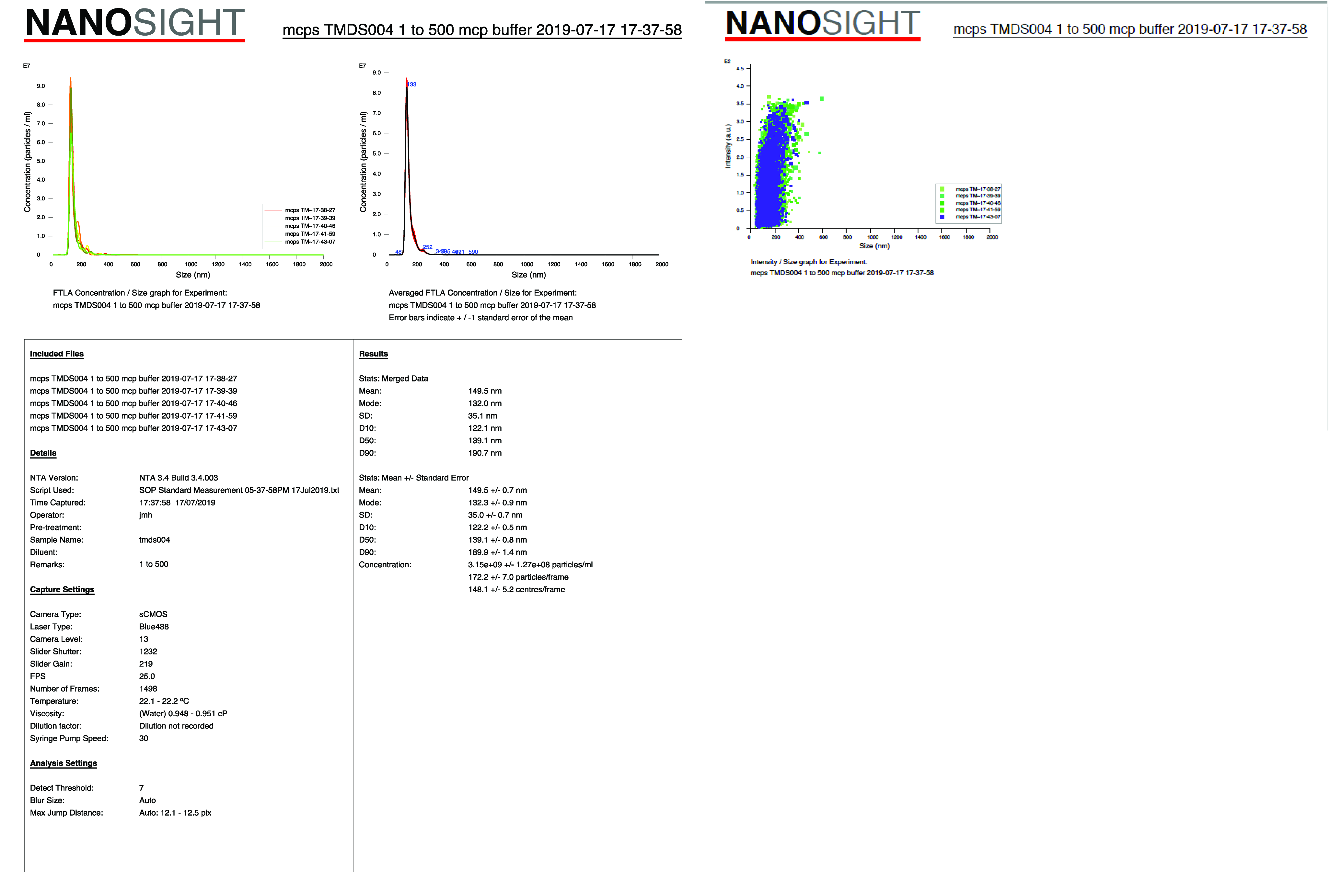

Supplement: S3 Fig — (TIF) [file pone.0226395.s004.tif]

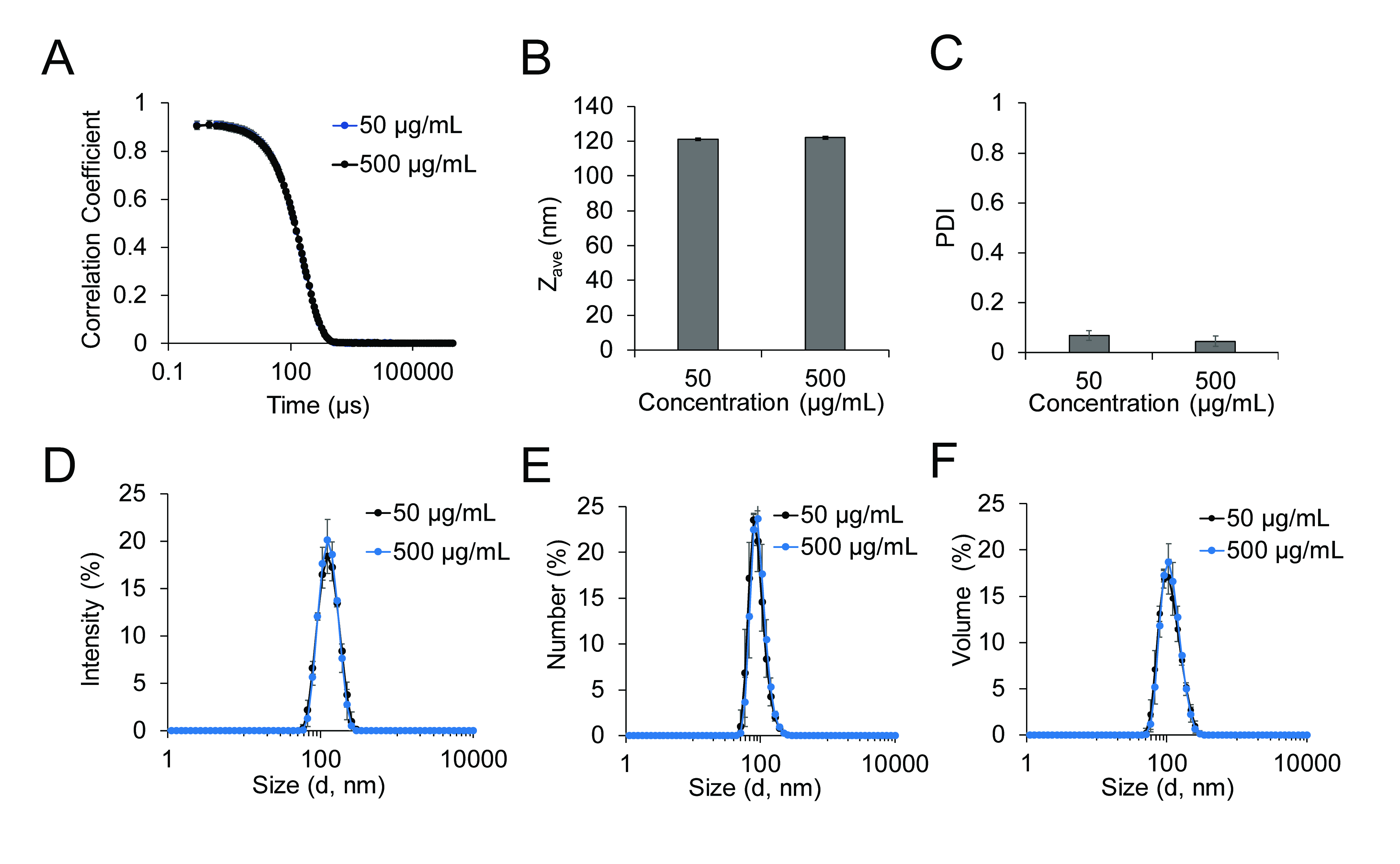

Supplement: S4 Fig — Raw correlation data (A), calculated Zave (B), and polydispersity indices (PDI) (C) of MCPs. Intensity (D), number (E), volume (F) particle size distributions of MCPs. (TIF) [file pone.0226395.s005.tif]
