# Supplementary material for: Apparent size and morphology of bacterial microcompartments varies with technique
Source: PLoS One. 2020 Mar 9;15(3):e0226395. doi: 10.1371/journal.pone.0226395 (PMC7062276; doi:10.1371/journal.pone.0226395)
Supplement: S2 File — (PDF) [file pone.0226395.s007.pdf]

S2 File: Calculation of Average Measured Diameter of a Sphere

We wish to relate the average diameter of a sphere measured by ultra-thin sectioning to the actual diameter of a spherical particle. To calculate the average measured diameter, D_{measured} of a sphere for which we know the actual diameter, D_{actual}, we use the equation for averaging a function over a volume.

$$f_{\text{avg}} = \frac{1}{Vol} \int_V f dV \tag{1}$$

In our case, we average over the function that defines the cross-sectional diameter of the sphere at any distance from the sphere's center, $d(r)$ (**S2 Fig**). This function can be defined as

$$d(r) = 2\sqrt{\left(\frac{D_{\text{actual}}}{2}\right)^2 - r^2} \tag{2}$$

Realizing that the volume we are integrating over is a sphere, we can write

$$Vol = \frac{4\pi}{3}\left(\frac{D_{\text{actual}}}{2}\right)^3 \tag{3}$$

Finally, we use spherical coordinates to evaluate the volume integral, yielding the expression

$$D_{\text{measured}} = \frac{6}{\pi D_{\text{actual}}^3} \int_0^{\pi} \sin(\phi) d\phi \int_0^{2\pi} d\theta \int_0^{D_{\text{actual}}/2} 2\sqrt{\left(\frac{D_{\text{actual}}}{2}\right)^2 - r^2} \; r^2 dr \tag{4}$$

Which simplifies to

$$D_{\text{measured}} = \frac{48}{D_{\text{actual}}^3} \int_0^{D_{\text{actual}}/2} \sqrt{\left(\frac{D_{\text{actual}}}{2}\right)^2 - r^2} \; r^2 dr \tag{5}$$

$$= \frac{3}{8} D_{\text{actual}} \left[\arcsin\left(\frac{2r}{D_{\text{actual}}}\right) - \frac{1}{4}\sin\left(4\arcsin\left(\frac{2r}{D_{\text{actual}}}\right)\right)\right]_{r=0}^{r=D_{\text{actual}}/2} \tag{6}$$

$$= \frac{3\pi}{16} D_{\text{actual}} \tag{7}$$
