# Supplementary material for: Apparent size and morphology of bacterial microcompartments varies with technique
Source: PLoS One. 2020 Mar 9;15(3):e0226395. doi: 10.1371/journal.pone.0226395 (PMC7062276; doi:10.1371/journal.pone.0226395)

Technical Replicate 1 20181016

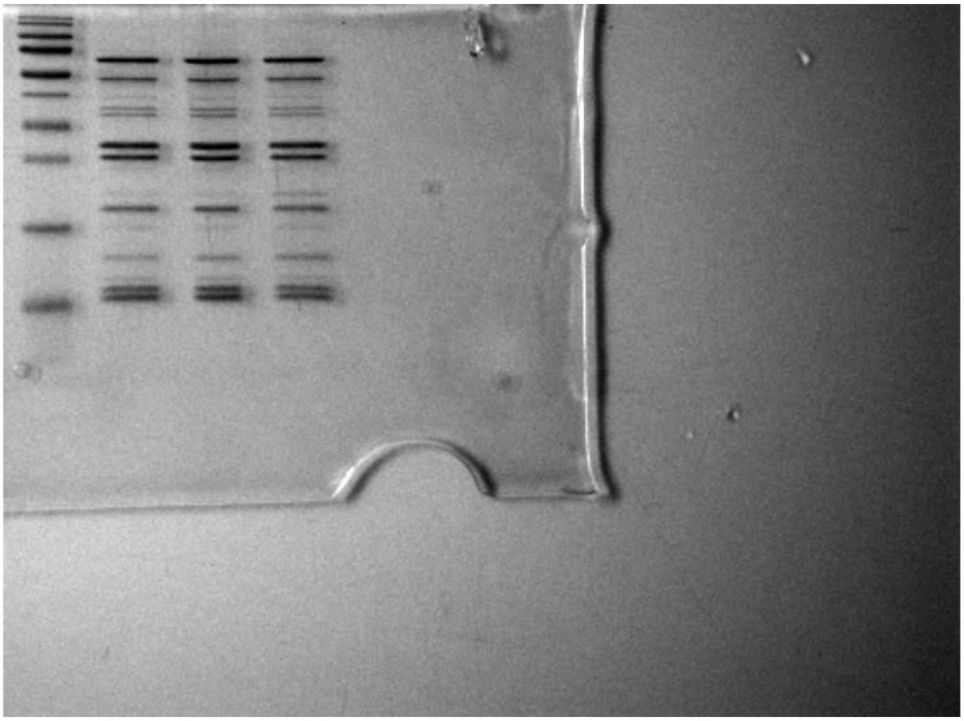

Technical Replicate 2 20181016

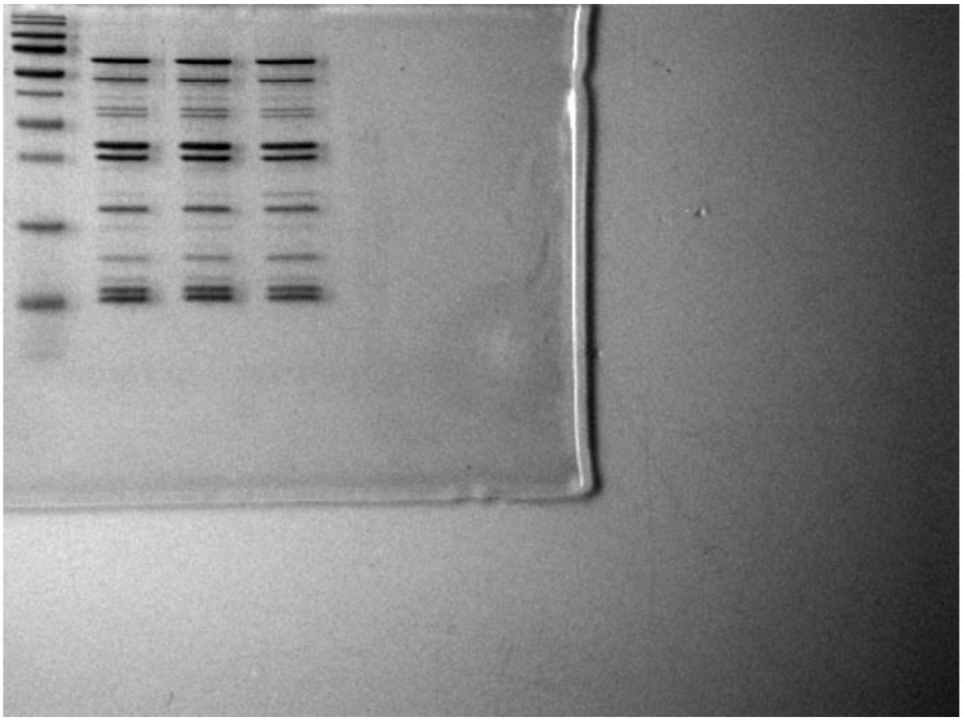

Technical Replicate 1 20181113

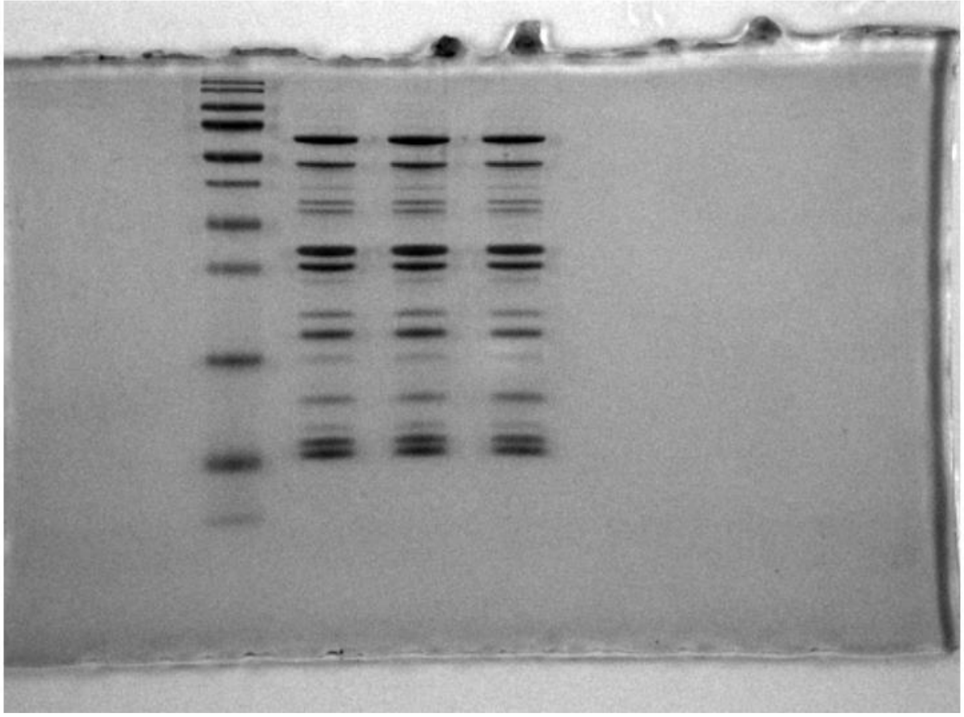

Technical Replicate 2 20181116

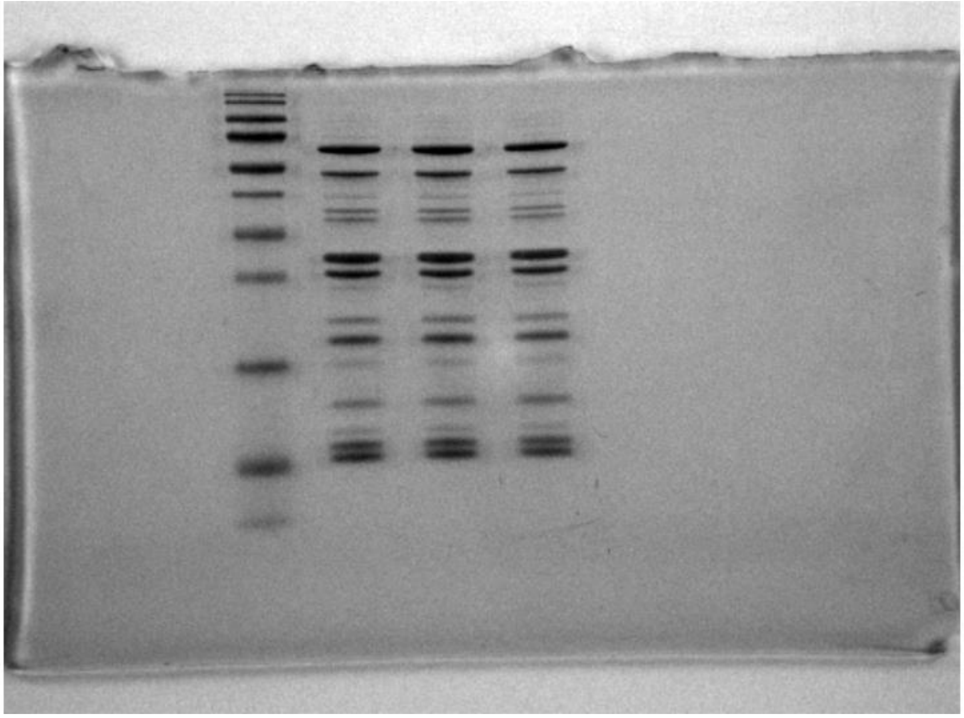

Coomassie 20190208

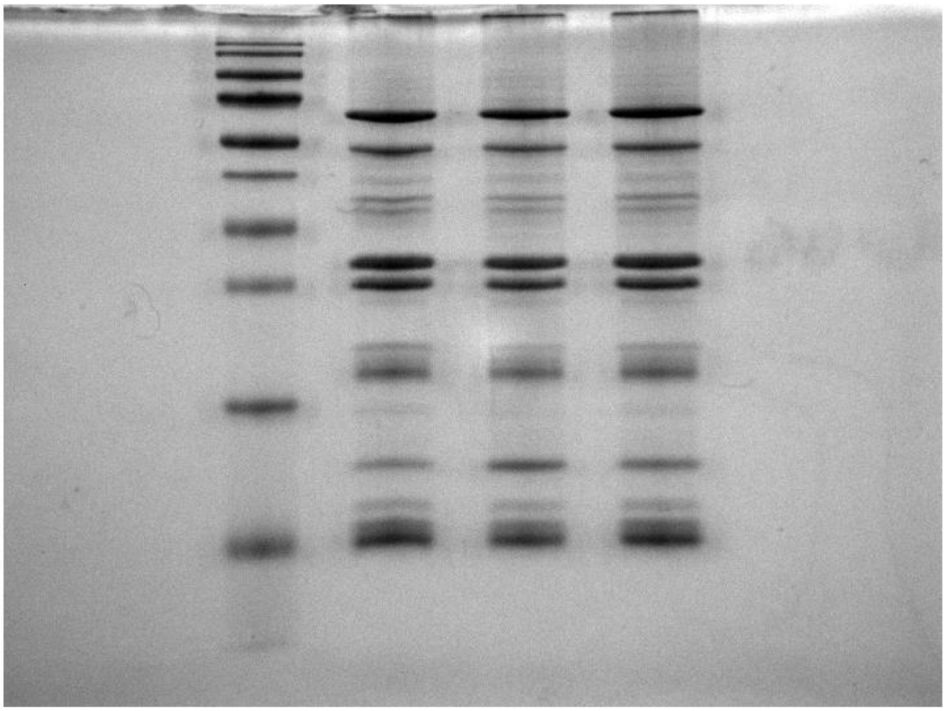

Supplement: S5 File — (PDF) [file pone.0226395.s010.pdf]
